# Involvement of SWAP-70 in proteolipid protein-induced experimental autoimmune encephalomyelitis

**DOI:** 10.55730/1300-0144.6161

**Published:** 2025-11-06

**Authors:** Canan ULUSOY, Gizem KORAL, Fatmanur AKPUNAR SALMAN, Melis ŞEN, Emine ŞEKERDAĞ KILIÇ, Recai TÜRKOĞLU, Cem İsmail KÜÇÜKALİ, Yasemin GÜRSOY-ÖZDEMIR, Vuslat YILMAZ, Erdem TÜZÜN

**Affiliations:** 1 Department of Neuroscience, Aziz Sancar Institute of Experimental Medicine, İstanbul University, İstanbul, Turkiye; 2 Research Center for Translational Medicine (KUTTAM), Koç University, İstanbul, Turkiye; 3 Department of Neurology, İstanbul Haydarpaşa Numune Training and Research Hospital, İstanbul, Turkiye; 4 Department of Neurology, Koç University, İstanbul, Turkiye

**Keywords:** Multiple sclerosis, experimental autoimmune encephalomyelitis, SWAP-70, follicular B cell, autoimmunity

## Abstract

**Background/aim:**

Multiple sclerosis (MS) is a chronic demyelinating inflammatory disease of the central nervous system. Studies have shown that B cells may play an important role in the induction and progression of MS. Switch-associated protein 70 (SWAP-70) is a signal transduction molecule abundantly expressed in B cells and involved in B-cell polarization, directed migration, endothelial cell adhesion, and tissue homing. B-cell stimuli increase its expression. SWAP-70 has been implicated in the pathogenesis of autoimmune disorders, including MS. This study aims to investigate the effects of acquired SWAP-70 inhibition on immune cell subsets in experimental autoimmune encephalomyelitis (EAE).

**Materials and methods:**

EAE was induced in Swiss James Lambert mice by immunization with proteolipid protein. EAE-induced mice were treated with either SWAP-70 short hairpin ribonucleic acid (shRNA) or nontargeting scrambled shRNA. Control PLP-immunized and non-PLP-immunized mice received saline treatment. The model was established by immunofluorescence studies demonstrating spinal cord demyelination and immune cell infiltration. Ratios of lymph node cell subsets were assessed by flow cytometry at termination.

**Results:**

All PLP-immunized mice showed clinical signs of EAE and demyelination and CD8+ T-cell/macrophage infiltration at spinal cord sections. SWAP-70 shRNA-treated mice showed significantly higher average clinical EAE scores than the other groups. SWAP-70 shRNA-treated mice showed significantly higher lymph node CD19+CXCR5+CD21+ follicular B-cell ratios than other groups, whereas ratios of other effector B-cell and T-cell subsets were comparable among groups.

**Conclusion:**

SWAP-70 appears to have an autoimmunity-suppressing effect in the PLP–EAE model, likely mediated by inhibiting follicular B cells. These findings propose a novel mechanism by which SWAP-70 might be involved in autoimmunity and endorse SWAP-70 as a potential target in novel MS-treatment strategies.

## 1. Introduction

Multiple sclerosis (MS) is a chronic demyelinating inflammatory disease of the central nervous system (CNS) caused by intricate interactions between autoreactive lymphocytes and innate immunity cell subsets. MS had been known as a T-cell-mediated autoimmune disease, but increasing evidence showed that B cells might play an important role in the induction and progression of MS [1]. The positive, beneficial effects of monoclonal antibodies targeting CD20+ B cells in MS provide further support for this notion [2].

In alignment with the significance of B cells in MS physiopathology, in a recent study focused on the microarray analysis of peripheral blood cells, we found that expression levels of a group of genes with functional relevance to B cells, including *BLK*, *BLNK*, *BANK1*, *FCRL2*, and switch-associated protein 70 (SWAP-70), were significantly altered in patients with MS. In the same study, the correlation network plot showed moderate correlations between SWAP-70 expression levels and peripheral blood effector B-cell ratios, as well as between SWAP-70 expression levels and sleep functions in MS patients [3].

In an independent study performed by a different cohort, peripheral blood SWAP-70 expression was shown to be increased during MS attacks and to be negatively correlated with the EDSS of the patients. Moreover, SWAP-70 expression in CD3-stimulated lymphocytes correlated with IL-6, IFN-γ, and IL-17 production, supporting the involvement of SWAP-70 in lymphocyte function in MS [4].

SWAP-70 and Def6 are two members of the SWEF family of signal transduction molecules that are abundantly expressed in B cells. SWAP-70 is involved in B-cell polarization, directed migration, endothelial cell adhesion, and tissue homing, and its expression is increased by B-cell stimuli [5]. Both SWEF members restrain IL-21 production; thus, the inborn deficiency of both SWEF proteins leads to increased IL-21 production by follicular T helper cells, increased B-cell activity, and follicular T helper cell expansion [6,7].

The significance of SWAP-70 in autoimmunity has been relatively understudied. In a couple of studies, SWAP-70 has been identified as a susceptibility locus for rheumatoid arthritis [8,9]. Notably, SWAP-70 and Def6 double knockout (KO) mice develop a lupus-like syndrome characterized by aberrant activity of autoantibody-producing B cells [10]. This dysregulation of B-cell activity was associated with enhanced IL-21 stimulation [6,7]. In a recent proteome-wide screening of 14 autoimmune disorders, SWAP-70 was found to be associated with rheumatoid arthritis but not with any other autoimmune disorders, including type 1 diabetes [9], suggesting that SWAP-70 does not appear to participate in common pathways across different autoimmune disorders.

Overall, these results suggest that SWAP-70 may constitute an attractive molecular target for novel therapeutic strategies in MS. However, our understanding of the impact of SWAP-70 on immune system functions is limited to KO animal studies. To assess the therapeutic value of this molecule, more information is needed on the immune system alterations caused by the acquired, transient deficiency of SWAP-70. Thus, to evaluate the impact of transient suppression of SWAP-70 on MS physiopathology, we investigated alterations in lymph node cell subsets in SWAP-70 short hairpin ribonucleic acid (shRNA)-treated adult mice using an experimental autoimmune encephalomyelitis (EAE) model.

## 2. Materials and methods

### 2.1. Mice and induction of EAE

To investigate the involvement of SWAP-70 in EAE induction, we utilized the relapsing–remitting EAE model induced by proteolipid protein (PLP) immunization in Swiss James Lambert (SJL) mice [11,12]. SJL mice were bought from the Jackson Laboratory (Bar Harbor, ME, USA) and maintained in pathogen-free conditions at the institutional animal research facility. The PLP–EAE model was generated by immunizing six-week-old female SJL mice with PLP139-151 (Peptides International, Louisville, KY, USA). The mice used in this study were sourced from the İstanbul University Animal Research Facility and housed and cared for in accordance with the Institutional Animal Care and Use Committee guidelines. These procedures were approved under decision no. 292762 by the İstanbul University Ethics Review Committee.

The study consisted of mice immunized with PLP and treated with saline (n = 7), SWAP-70 shRNA (n = 7), nontargeting scrambled shRNA (n = 7), and non-PLP-immunized mice (n = 7). PLP139-151 was dissolved in PBS with a 1 mg/ml stock concentration. Within a total volume of 200 μL, 100 μg of PLP139-151 peptide per mouse was emulsified with Complete Freund’s Adjuvant (CFA) (1:1) containing 533 μg desiccated *Mycobacterium tuberculosis* H37Ra. The total dose of 200 μL was divided into 4 areas and subcutaneously (s.c.) injected into mice under isoflurane anesthesia. One hour after immunization, 250 ng of pertussis toxin (PTX) was also administered intraperitoneally (i.p.) per mouse. Control non-PLP immunized mice received s.c. CFA (without PLP), i.p. PTX and i.p. saline treatment. SWAP-70, scrambled shRNA, and saline treatments were administered 1 day after the immunization. Evaluation of EAE clinical findings included clinical EAE scoring of the tail and limbs (scales 0–5) [13] by a blinded investigator: 0 = Healthy, 1 = Flaccid tail, 2 = Ataxia or paralysis of the hind limbs, 3 = Paralysis of the hind limbs and/or ataxia or paralysis of the forelimbs, 4 = Ataxia or paralysis of all limbs, 5 = Moribund. On day 20 after the immunization, mice were sacrificed, and spinal cord and spleen samples were collected.

### 2.2. shRNA treatment

To silence the SWAP-70 gene, a lentiviral particle carrying shRNA (sc-153963-V, Santa Cruz Biotechnology, Dallas, TX, USA) was purchased. A nontargeting scrambled shRNA (Plasmid-A sc-108060, Santa Cruz Biotechnology, Dallas, TX, USA) was selected as a control. After the toxicity tests, the dosage of 100,000 IFU was chosen for shRNA treatment. Mice were randomly assigned to treatment arms using a random number generator. A single dose of shRNA (100,000 infection-forming units [IFUs] in 100 μL saline) was administered i.p. Saline-treated groups received only 100 μL of saline. No apparent adverse effects (e.g., weakness, decreased mobility, death) were observed following the shRNA injections.

### 2.3. Quantitative real-time polymerase chain reaction (RT–PCR)

To confirm successful silencing of the SWAP-70 gene by shRNA, RNA was isolated from frozen spleen cells obtained at the time of mouse termination (20 days after immunization). RNA extraction was performed according to the kit protocol (Qiagen, Hilden, Germany). The quality of the obtained RNAs was measured and stored at – 80°C until complementary deoxyribonucleic acid (cDNA) synthesis, which was performed using the High-Capacity cDNA Reverse Transcription Kit (Applied Biosystems, Waltham, MA, USA). cDNAs were synthesized by a Thermal Cycler device (Bio-Rad, Hercules, CA, USA) with the synthesis plan designed to be kept at 25 °C for 10 min, at 37 °C for two h, and at 65 °C for 5 min. The *RPLP0* gene (Forward: GTTCTTTGGGGAGCCAACAG, Reverse: GCTCCCTGGTTTCTCTTCCT), which is highly expressed in mice, was used as the reference gene. Messenger RNA (mRNA) levels of SWAP-70 were determined by using commercial primers (Forward: CAGTAGCTGGCAGGTGTATTAG, Reverse: TGGAGTCTTCTGGTGTAATGTG) (Qiagen). RT–PCR was performed on the CFX96 Touch Real-Time PCR device (Bio-Rad, Hercules, CA, USA), using the LightCycler 480 SYBR Green I Master kit (Roche, Basel, Switzerland) according to the manufacturer’s instructions. Samples were run in duplicate for all genes. Data were analyzed according to the ΔΔCt method, and the results were expressed as relative mRNA (2^- (ΔΔCT)) levels. SWAP-70 shRNA-administered mice exhibited an average (± standard deviation) spleen SWAP-70 gene expression level of 4.4 ± 0.8, whereas saline-treated and scrambled shRNA-treated mice showed expression levels of 12.7 ± 1.8 and 14.8 ± 3.4, respectively. This corresponded to an approximate 68% reduction in splenic SWAP-70 expression levels after single shRNA administration.

### 2.4. Immunofluorescence staining for spinal cord demyelination and immune cell infiltration

At termination, mice were perfused with 4% paraformaldehyde (PFA), and spinal cord samples were isolated and stored in 4% PFA for 24 h. The samples were then dehydrated using a sucrose gradient (10%, 20%, and 30%) in 0.1 M phosphate buffer at 4 °C and embedded in Cryomatrix resin (Thermo Fisher Scientific, Waltham, MA, USA). Tissues were sliced at a thickness of 10 microns per section. Slides were washed three times in Dulbecco’s Phosphate-Buffered Saline (DPBS) and then blocked with Super Block (Thermo Fisher Scientific) solution for one h at room temperature. Next, after removing the blocking solution, tissue sections were incubated with rat antimyelin basic protein (MBP, Abcam, Cambridge, UK), rat anti-F4/80 (Abcam) for macrophages, or rabbit anti-CD8 (Abcam) for cytotoxic T cells for 1.5 h at 37 °C. The antibody solution was removed, and the slides were washed three times with DPBS for 5 min. Then, the tissue sections were incubated with appropriate secondary antibodies diluted in blocking solution (Abcam) for 1.5 h at 37 °C in the dark. Subsequently, the secondary antibody was removed, and sections were washed in DPBS. Next, tissue sections were mounted with 4’, 6-diamidino-2-phenylindole (DAPI) containing mounting medium and observed by fluorescence microscopy.

The density of T cells, macrophages, and demyelination areas was semiquantified using software. For this purpose, 30 spinal cord sections per mouse were utilized for each parameter. Fluorescence microscopy images exported as TIFF files from the LAS X program (Leica, Germany) were imported and analyzed with ImageJ 1.52e (National Institutes of Health, USA). For cell counting, DAPI-positive cells were marked with “Find Maxima”. The noise was adjusted to keep each cell as a single point. The results were expressed as cell count/mm^2^. Fluorescent staining of MBP was represented in integrated density (per μm^2^). Detailed image analysis is described elsewhere (12).

### 2.5. Flow cytometry

Lymph node cells were used for immunophenotyping of major T-cell and B-cell populations. Frozen cells were thawed in a water bath set at 37 °C and centrifuged in medium (Roswell Park Memorial Institute [RPMI] 1640 and 10% fetal bovine serum [FBS]) at 1800 rpm at +4 °C for 10 min. Cells were stained with antimouse CD25-PE, CD3-PerCP/Cy5.5, CD8a-APC, CD4-APC/Cy7, CD1d-PerCP/Cy5.5, CD19-APC, CD38-APC/Cy7, CXCR5-FITC, and CD21-APC/Cy7 (BioLegend, San Diego, CA, USA) for 30 min at room temperature in the dark. After washing, the cells were centrifuged and resuspended in PBS. BD FACSAria II (Becton, Dickinson and Company, Franklin Lakes, NJ, USA) was used for flow cytometry. Analyses were performed using FlowJo (Becton, Dickinson & Company, Version 10.8.1) software.

Gating strategy is a supplementary issue.

### 2.6. Statistical analysis

A repeated-measures analysis of variance (ANOVA) was employed to compare clinical grades. Immunological variables were compared with ANOVA and Tukey’s post hoc test. Any p-values less than 0.05 were considered statistically significant.

Correlation analyses were performed using Pearson’s correlation coefficient to assess the relationships among immune cell frequencies, MBP areas, and clinical scores.

## 3. Results

### 3.1. SWAP-70 shRNA-treated mice show increased EAE severity

**Figure 1:**
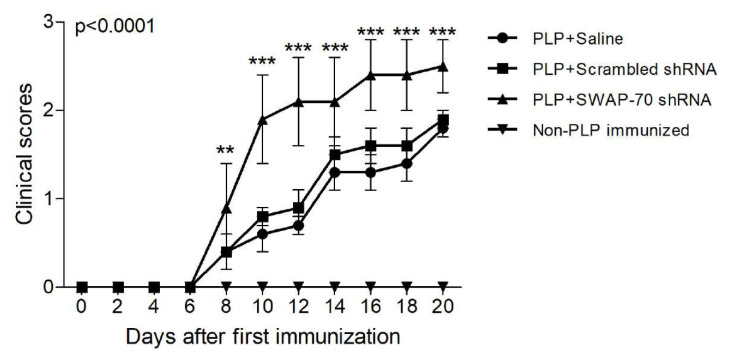
Kinetics of EAE scores for different shRNA treatment arms and control mice after the PLP immunization. The p-value shown in the upper left corner of the panel was obtained by ANOVA. **, p < 0.01; ***, p < 0.001. Vertical bars denote standard deviations.

Following immunization with PLP in CFA and PTX, all immunized mice developed muscle weakness from day 6 onward. Average clinical scores of SWAP-70 shRNA-treated mice were higher than those of other treatment arms between days 8 and 20, culminating in significantly enhanced EAE severity at termination (p < 0.0001) in the SWAP-70 shRNA-treated mouse group. None of the non-PLP immunized mice showed clinical signs of EAE (Figure 1).

**Figure 2:**
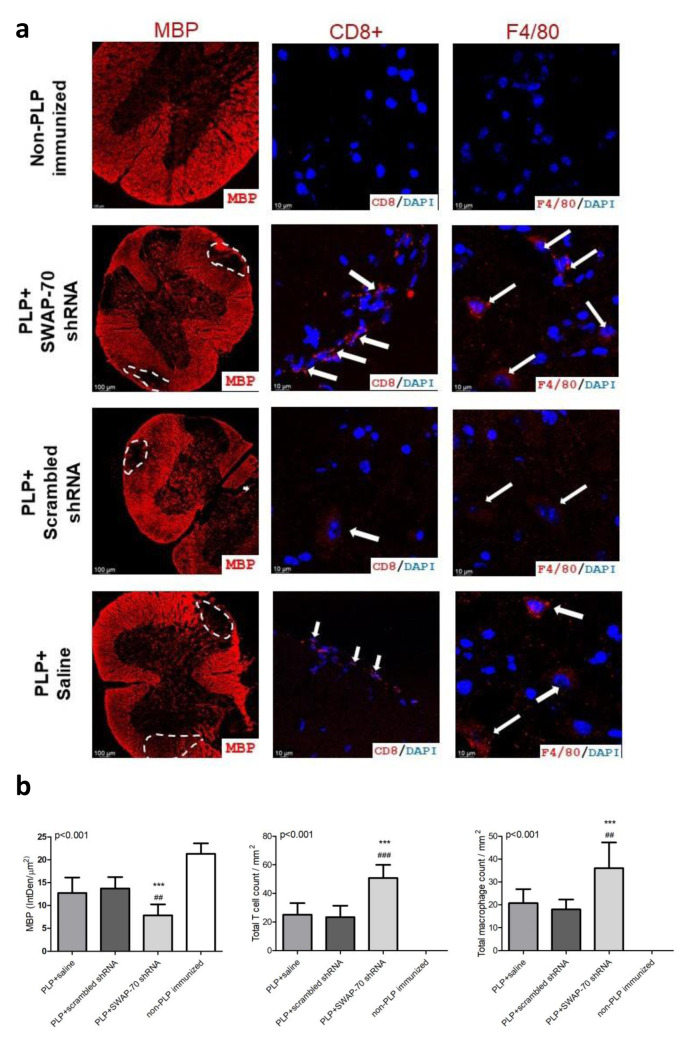
Analysis of EAE lesions in spinal cord sections through semiquantification of the intensity of CD8+ T cell, F4/80+ macrophage infiltration, and demyelination areas. a) Ten-micron spinal cord tissue sections of mice belonging to different shRNA treatment arms and control groups were stained for myelin basic protein (MBP), CD8+ T cells, and F4/80+ macrophages. b) Total cell count is shown per mm^2^ area, and the expression levels of MBP are shown as integrated density (IntDen) per μm^2^ area. Error bars indicate the standard deviations. The p-values obtained by ANOVA are denoted in the upper left corner of the panel. Significantly different by pair-wise comparison at: ***p < 0.001 for comparisons between SWAP-70 shRNA-treatment versus non-PLP immunized and ##p < 0.01, ###p < 0.001 for comparisons between SWAP-70 shRNA-treatment versus saline and scrambled shRNA treatment arms.

Image analysis of spinal cord sections exhibited significantly reduced MBP+ areas (thus increased demyelination) (Figure 2a). It significantly increased CD8+ T cell and F4/80+ macrophage counts (Figure 2b) in the SWAP-70 shRNA-treated mouse group compared with the other three groups. There were no significant differences between the saline and scrambled shRNA-treated groups in MBP+ areas or in T-cell and macrophage infiltration intensities (Figures 2a, 2b).

Moreover, strong correlations were observed between clinical scores and the frequency of MBP^+^ areas (R = −0.700, p < 0.001).

### 3.2. Impact of SWAP-70 shRNA-treatment on effector and regulatory lymphocyte populations

**Figure 3:**
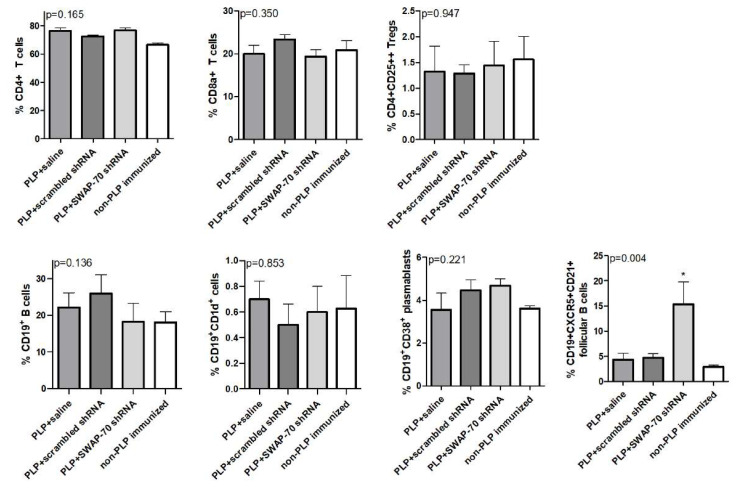
Populations (%) of lymph node cell subtypes of proteolipid protein-immunized and control mice. Error bars indicate the standard deviations. The p-values obtained by ANOVA are denoted in the upper left corner of the panel. Significantly different by pair-wise comparison at: *p < 0.05.

To assess the impact of SWAP-70 shRNA treatment on major immune cell subtypes, we assessed the distribution of major effector and regulatory T cell (Sup. Figure 1), B-cell and follicular B-cell populations (Supplementary Figure 2) in the lymph node cells of PLP-immunized and control mice by flow cytometry. All study groups showed comparable CD4+ T cell, CD8a+ T cell, and CD4+CD25++ regulatory T cell (Treg) ratios. Similarly, the ratios of CD19+ B cells, CD19+CD38+ plasmablasts, and CD19+CD1d+ B cells were identical across groups. As an exception, SWAP-70 shRNA-treated mice showed significantly higher CD19+CXCR5+CD21+ follicular B-cell ratios than saline-treated, scrambled shRNA-treated, and non-PLP-immunized mice (Figure 3).

### 3.3. Correlation of clinical scores and immune cells

Correlation analysis revealed a significant association between the frequency of follicular B cells (CD19^+^CXCR5^+^CD21^+^) and clinical scores (R = 0.654, p = 0.003). Moreover, strong correlations were observed between clinical scores and the frequencies of T cells (R = 0.787, p < 0.001) and macrophages (R = 0.768, p < 0.001). These findings suggest a close relationship between immune cell dynamics and disease severity.

## 4. Discussion

In this study, we substantially suppressed SWAP-70 expression for less than 3 weeks with a single shRNA administration. This has culminated in a significant worsening of EAE symptoms, thus suggesting that SWAP-70 has an autoimmunity-suppressing action in the PLP–EAE model. Our results align with experimental animal model studies showing increased susceptibility to lupus-like disease in SWAP-70 KO mice and underscore that inborn and acquired deficiencies of SWAP-70 are equally hazardous for autoimmunity induction [6,7]. Our results also align with previous clinical MS studies showing increased clinical severity (EDSS score) in patients with lower peripheral blood SWAP-70 gene expression levels [4]. We place special emphasis on B-cell subsets because SWAP-70 is abundantly expressed in B cells [5] and its expression correlates with peripheral blood subset ratios in MS [3]. Our findings suggest that at least one factor underlying enhanced disease activity in EAE-induced and shRNA-treated mice is follicular B cells. This assertion is consistent with the high expression of SWAP-70 in the follicular cell region of lymph node germinal centers and follicular lymphomas [14].

Follicular B cells reside in lymphoid follicles containing germinal centers. They are involved in primary immune responses with the assistance of follicular T helper cells, thereby establishing antibody isotype switching and memory B-cell formation [15,16]. In the EAE model, follicular B cells have been shown to reside and participate in disease mechanisms not only in lymph nodes but also in meningeal ectopic lymphoid follicles [17]. Thus, it is conceivable that increased ratios of follicular B cells might contribute to enhanced disease activity in SWAP-70-suppressed mice. Furthermore, ongoing clinical trials have demonstrated that anti-CD19 therapies and Bruton’s tyrosine kinase inhibitors can effectively modulate B-cell activity, offering new avenues for treatment in MS patients, including reducing lesion load in the brain, reducing relapse rates, and potentially slowing disease progression [18].

SWAP-70 has been shown to participate in migration and vascular adhesion of immune cells [5,19]. Our study suggests that an additional function of SWAP-70 might be the suppression of excessive follicular B-cell activity and, thus, the inhibition of potential autoimmune reactions before they start. Notably, inhibition of SWAP-70 expression by shRNA did not appear to influence major effector T-cell and B-cell subsets in our study, suggesting that transient acquired suppression of SWAP-70 does not interfere with major immune cell subsets required for host defense against infectious microorganisms. This feature contrasts with the effects of monoclonal antibodies targeting B cells (e.g., ocrelizumab and rituximab), which deplete a wide range of B cells and thus impair host defense in both MS and EAE. [20,21].

In a single study, SWAP-70 KO mice have shown reduced ratios of marginal zone B cells, characterized by high CD1d expression [22]. SWAP-70 shRNA failed to suppress CD1d+ B cells in our study, suggesting that marginal zone B cells are not affected by transient or acquired SWAP-70 inhibition. That suppression of this cell subset requires inborn, long-term inhibition of SWAP-70 activity.

In contrast with our study, mice KO for the alternative SWEF molecule Def6 have been shown to be resistant to EAE induced by myelin oligodendrocyte glycoprotein immunization [23]. This finding aligns with the notion that, while Def6 and SWAP-70 appear to perform similar roles in autoantibody production, they actually have divergent functions. Def6 KO mice show reduced germinal center activity and preserved mature B-cell formation, whereas SWAP-70 KO mice show preserved germinal center activity and altered plasma cell formation. Secondly, SWAP-70 is primarily responsible for B-cell functions, whereas Def6 is also involved in T helper cell activity [6]. Specifically, Def6 KO mice exhibit suppressed Th1 and Th17-type T helper activity, which is crucial for EAE induction [24]. These discrepancies may explain the diverse EAE susceptibility in Def6 KO and SWAP-70 shRNA-treated mice.

The suppressive role of SWAP-70 in autoimmunity had previously been established in a lupus-like animal model [5]. Therefore, since we did not anticipate a disease-ameliorating effect, we did not conduct a treatment study and thus did not initiate shRNA treatment after EAE was established. Since our aim was to determine whether SWAP-70 inhibition exacerbates EAE and identify potential mechanisms underlying this potential exacerbation, we chose to perform a prevention study and suppress SWAP-70 expression before the onset of full-scale EAE.

Despite the promising outcomes, our study has several limitations that warrant consideration in future research. Notably, we did not perform complementary analyses of secondary lymphoid tissues, such as the spleen, which could provide additional insight into B-cell and T-cell compartmentalization and functional dynamics [11,12,15]. Furthermore, cytokines play a pivotal role in lymphocyte activation, differentiation, and class switching, and their quantification in serum, lymph nodes, and other relevant compartments would be essential for a more comprehensive understanding of immune modulation [5, 6]. Another limitation is that our validation relied exclusively on mRNA-level measurements, and confirmation at the protein level would provide a more robust assessment of the observed molecular changes [3, 4]. Finally, the sample size was limited, and the study was designed as a preventive rather than therapeutic investigation; future studies employing larger cohorts and therapeutic interventions will be crucial to determine the translational potential of these findings [11,12,18].

Our findings highlight SWAP-70 as a critical regulator of B-cell function in CNS autoimmunity. Future studies should explore the therapeutic potential of modulating SWAP-70 using microRNA (miRNA) mimics or antagomirs to fine-tune B-cell differentiation and trafficking. Integrating these strategies with in vivo models will be essential to assess their translational relevance.

## Supplementary Information

Supplementary Figure 1 B-cell gating strategy used to identify CD19^+^ B-cell subsets. Debris was excluded using FSC–SSC characteristics, followed by singlet selection (FSC-A vs FSC-H). Live lymphocytes were gated, and CD19^+^ B cells were defined as the parent population. Percentages displayed within each gate represent the proportion of cells relative to the immediate parent population.

Supplementary Figure 2 T-cell gating strategy used to identify major T-cell subsets. Debris was excluded based on FSC–SSC distribution, followed by singlet selection using FSC-A versus FSC-H. Live lymphocytes were gated, and CD3^+^ T cells were identified as the parent population. Within CD3^+^ cells, CD4^+^ T helper cells, CD8^+^ cytotoxic T cells, and CD4^+^/CD8^+^ double-positive (DP) cells were delineated. CD4^+^CD25^++^ regulatory T cells (Tregs) and CD4^+^CD27^+^ memory T cells were subsequently gated as downstream subsets. Percentages shown within gates represent the proportion of each population relative to its immediate parent population.
