# Supplementary figures and images for: Involvement of SWAP-70 in proteolipid protein-induced experimental autoimmune encephalomyelitis
Source: Turk J Med Sci. 2025 Nov 6;56(1):274–81. doi: 10.55730/1300-0144.6161 (PMC12974271; doi:10.55730/1300-0144.6161)

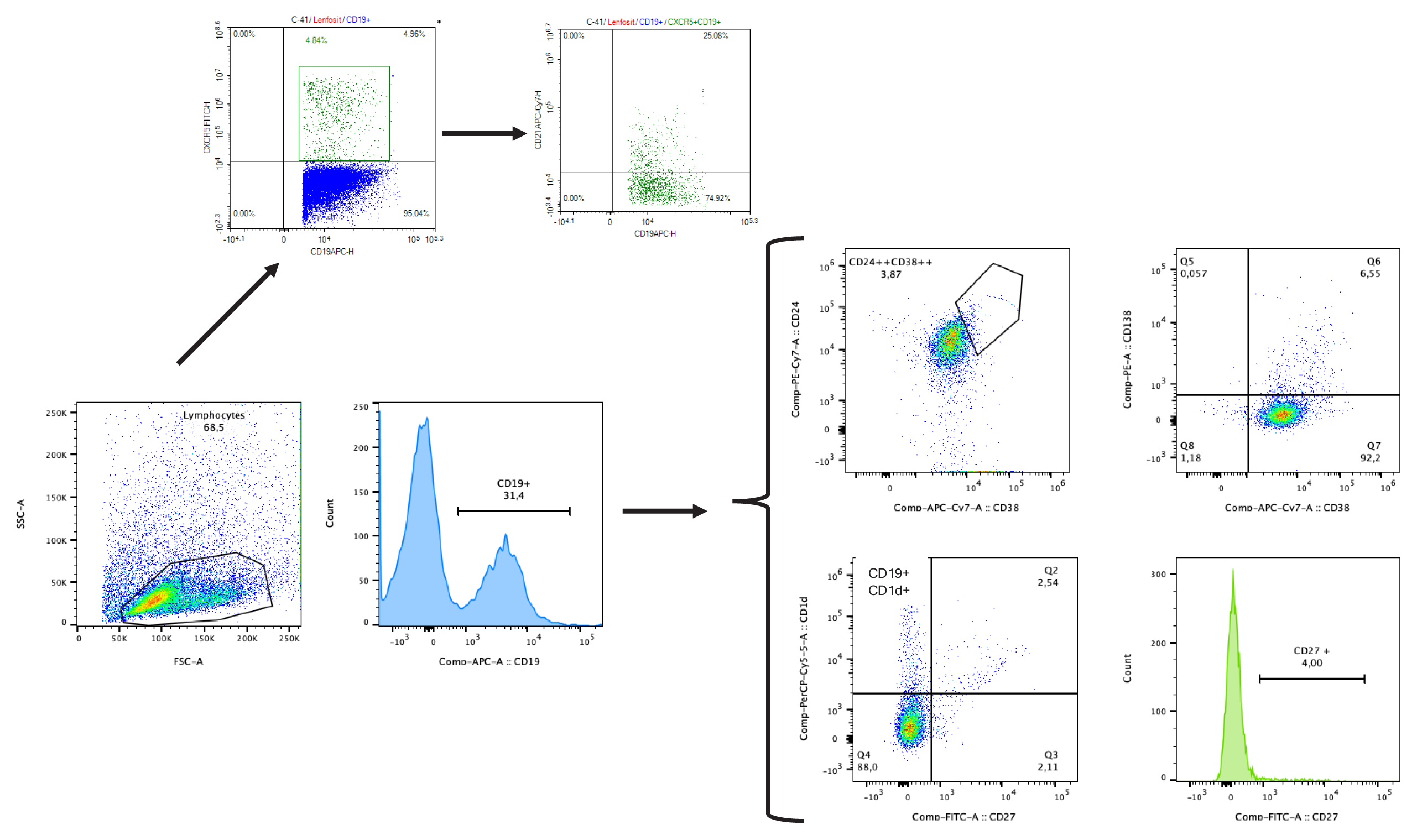

Supplement: Supplementary Figure 1 — B-cell gating strategy used to identify CD19+ B-cell subsets. Debris was excluded using FSC–SSC characteristics, followed by singlet selection (FSC-A vs FSC-H). Live lymphocytes were gated, and CD19+ B cells were defined as the parent population. Percentages displayed within each gate represent the proportion of cells relative to the immediate parent population. [file tjmed-56-01-274s1.tif]

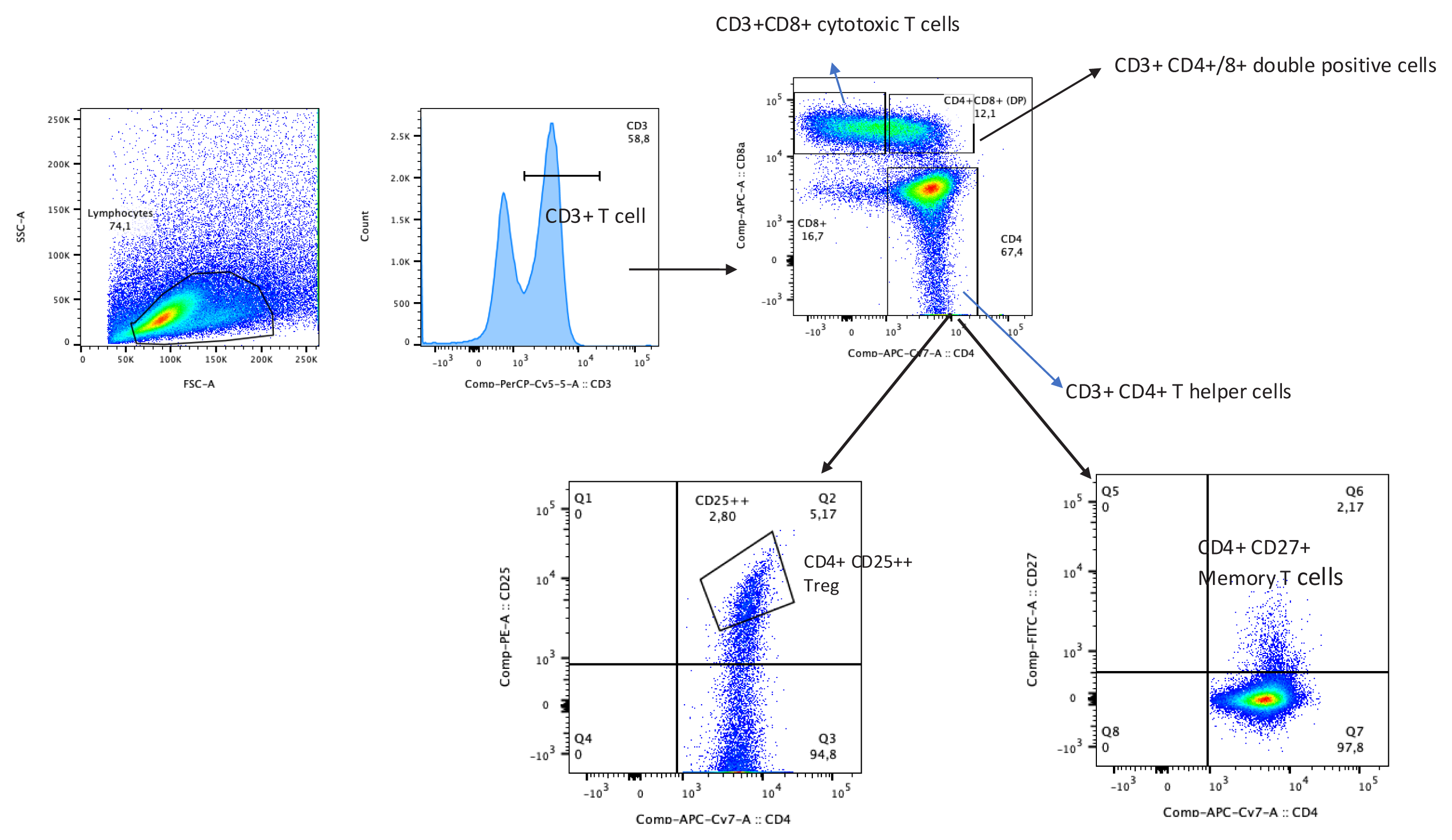

Supplement: Supplementary Figure 2 — T-cell gating strategy used to identify major T-cell subsets. Debris was excluded based on FSC–SSC distribution, followed by singlet selection using FSC-A versus FSC-H. Live lymphocytes were gated, and CD3+ T cells were identified as the parent population. Within CD3+ cells, CD4+ T helper cells, CD8+ cytotoxic T cells, and CD4+/CD8+ double-positive (DP) cells were delineated. CD4+CD25++ regulatory T cells (Tregs) and CD4+CD27+ memory T cells were subsequently gated as downstream subsets. Percentages shown within gates represent the proportion of each population relative to its immediate parent population. [file tjmed-56-01-274s2.tif]
